# Beyond muscle activation: the role of biomechanical properties of muscles in determining dynamic knee valgus in female volleyball players

**DOI:** 10.1186/s13102-026-01681-1

**Published:** 2026-04-16

**Authors:** Nizami Chalabiyev, Sibel Basaran, Volkan Deniz, Ümit Adaş, Aylin Sariyildiz

**Affiliations:** 1 Faculty of Medicine, Department of Physical Medicine and Rehabilitation, Cukurova University, Adana, Türkiye; 2 Faculty of Health Sciences, Department of Physiotherapy and Rehabilitation, Tarsus University, Mersin, Türkiye; 3 Faculty of Sports Science, Department of Coaching Education, Cukurova University, Adana, Türkiye

**Keywords:** Dynamic knee valgus, Electromyography, Muscle activity, Volleyball

## Abstract

**Background:**

Dynamic knee valgus (DKV) is considered a risk factor for knee injuries, particularly in female athletes. Muscle activation and biomechanical properties are thought to play a role in its development, yet their exact contributions remain unclear. This study aimed to investigate the biomechanical properties and electromyographic activity of lower extremity muscles in female volleyball players with and without DKV.

**Methods:**

Thirty-six female volleyball athletes aged 12–18 were allocated to DKV (*n* = 18) and control (*n* = 18) groups based on single-leg squat test. Biomechanical properties (muscle tone, elasticity, stiffness) of the muscles were measured using MyotonPro^®^. Surface electromyography was used to assess muscle activation and onset times of the gluteus medius (GM), adductor magnus (AM), vastus medialis (VM), vastus lateralis (VL), semimembranosus (HM), and biceps femoris (HL) during landing phase of the volleyball spike.

**Results:**

There were no significant demographic or sport-specific differences between the groups. VM/VL and HM/HL muscle tone ratios and AM/GM, VM/VL and HM/HL stiffness ratios were significantly higher in DKV group (*p* < 0.05). DKV group showed significantly lower activation in GM and HL and higher AM/GM and HM/HL ratios (*p* = 0.001 and *p* = 0.015). GM-AM onset time difference was significantly lower in DKV group (*p* < 0.001). Multiple linear regression analysis did not reveal any statistically significant predictors of knee valgus angle during the landing phase of the spike.

**Conclusion:**

The tone and stiffness ratios of the medial thigh muscles to the lateral ones (AM/GM and HM/HL) were increased. Activation of GM and HL muscles were decreased in athletes with DKV. Since neither biomechanical properties nor muscle activation affected frontal knee valgus angle during the spike, it is speculated that the change may reflect a musculoskeletal adaptation.

## Introduction

Volleyball is recognized as one of the most dynamic and popular sports, with the Fédération Internationale de Volleyball comprising more than 150 member countries and an estimated 200 million professional players worldwide [1]. Although volleyball is categorized as a non-contact sport in which two teams are separated by a net, the rapid movements, powerful jumps and landings, and the specific positional demands of the game render it a sport with a relatively high incidence of injuries [2]. Notably, approximately 50% of all musculoskeletal injuries in volleyball are reported to occur at the knee joint [2]. Knee injuries are markedly more common in female athletes, with an incidence rate nearly 3.5 times higher than that observed in male athletes [3].

Dynamic knee valgus (DKV), visually observed as medial knee displacement [4] is considered an important biomechanical predisposing factor for knee injuries in female athletes [5, 6]. In volleyball players, DKV typically occurs during the landing phase of a spike or blocking [7]. Previous electromyography (EMG) studies have demonstrated that gluteal muscle activation [8], vastus medialis and vastus lateralis activity [9], hamstring and gastrocnemius recruitment [10], as well as the agonist–antagonist activation ratio may represent key kinetic parameters in determining DKV [11]. In studies conducted on volleyball players with DKV, muscle activation patterns have most commonly been examined under experimental conditions during single- or double-leg jumps from a standardized height [12, 13], or during double- to single-leg squats [14]. However, observations obtained in the complex environment of the sport may differ from those in controlled laboratory settings. Tasks such as the single-leg squat, bilateral or unilateral drop jumps, and other laboratory-based assessments are typically performed in a controlled, predictable, and relatively slow manner, allowing for high reproducibility but limited ecological validity [8, 11]. In contrast, spike-landing is a highly dynamic, sport-specific action that includes a rapid multi-step approach, a forceful vertical jump, and reactive landing influenced by game-related situational demands. These features substantially increase the mechanical demands on the lower extremity and may lead to different lower extremity kinematics [15]. Notably, there is a lack of studies investigating muscle activations and recruitment timings during volleyball-specific complex movements, such as spike or blocking, where knee injuries frequently occur.

Alongside muscle activity, biomechanical properties of muscle may also influence segmental alignment in dynamic conditions. Biomechanical properties refer to passive muscle characteristics—such as tone, stiffness, and elasticity—that determine the intrinsic ability of muscles to generate passive tension around a joint and thereby influence segmental alignment during movement [16]. Previous studies have demonstrated that statically measured muscle biomechanical properties can affect athletic performance [16, 17] as well as certain kinematic parameters in athletes [18]. Considering previous findings [16–18] and the biomechanical perspective of DKV [5], the tone, stiffness, or elasticity of the hip adductors, tensor fasciae latae, and vastus medialis may differ from those of their respective antagonists in athletes with DKV. Such alterations could contribute to imbalanced passive tension around the hip and knee, thereby influencing frontal-plane knee alignment during dynamic tasks [16]. However, no study to date has demonstrated whether these biomechanical properties actually influence the development or presence of DKV. Only a previous study determined the normative values of lower extremity muscles in male participants with DKV using myotonometric assessment [19]. To the best of our knowledge, no study has investigated the relationship between muscle biomechanical properties and DKV in female volleyball players. Examining muscle activation impairments, and potential alterations in muscle biomechanical properties that may contribute to DKV in volleyball players, during sport-specific movements, could provide sports and health professionals with valuable insights for the prevention of knee injuries.

The objective of this study is to compare the muscle biomechanical properties of female volleyball players with and without DKV. Furthermore, it aims to determine the influence of muscle activation and biomechanical properties on the valgus angle in the frontal plane during the landing phase of a spike.

## Methods

### Study design

**Fig. 1:**
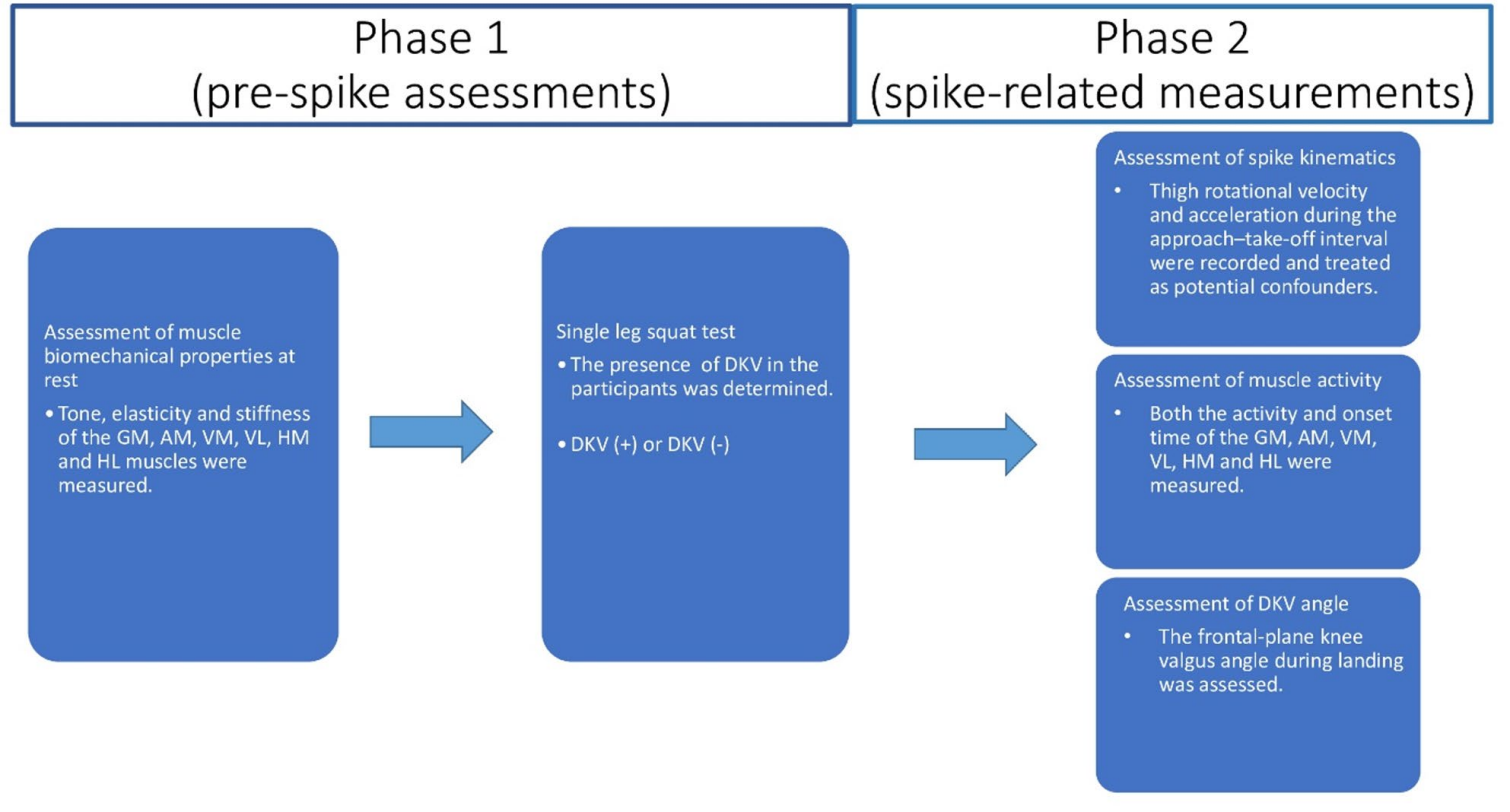
Experimental protocol of the study. Abbreviations: GM, Gluteus medius; AM, Adductor magnus; VM, Vastus medialis; VL, Vastus lateralis; HM, Semimembranosus; HL, biceps femoris; DKV, dynamic knee valgus

The study was reported in accordance with the Strengthening the Reporting of Observational Studies in Epidemiology checklist. This observational comparative was carried out with two phases between May 2024 and September 2024 at Cukurova University and Adana Kare Sports Club. In the first phase of the study, the biomechanical properties of the thigh muscles were compared between young female volleyball players with and without DKV. In the second phase, the effect of thigh muscle activation and biomechanical properties on the knee valgus angle during the landing phase of a volleyball spike was investigated (Fig. 1). Ethical approval was obtained from the Clinical Research Ethics Committee of Cukurova University (Decision No: 140/25, Approval Date: 04.01.2024). All participants and their legal guardians were fully informed about the study procedures, and written informed consent was obtained prior to participation. All procedures adhered to the principles of the Declaration of Helsinki.

### Sample size

The required sample size was calculated using G*Power software (version 3.0.18) (Heinrich-Heine-Universität Düsseldorf, Germany). Based on the study by Mauntel et al., a sample size sufficient to detect a significant difference in lower-extremity muscle activation ratios between the experimental and control groups was determined [4]. With an effect size of d = 0.97, a power of 80% (1-β = 0.80), and an alpha level of 0.05 (type I error), the minimum required sample size was estimated as 18 participants per group, for a total of 36 participants.

### Participants

Participants were recruited using a randomized invitation sequence. Each athlete registered to the Adana Kare Sports Club was assigned a unique identification number, which was then entered into a randomization software program (www.randomizer.org). Based on the randomized order generated, athletes were invited to join the study through verbal communication and text messaging. Recruitment proceeded in this manner until the target sample size was achieved. The inclusion criteria for the study were as follows: Female athletes aged between (i) 12 and 18 years, (ii) actively playing volleyball in a sports club for at least one year, and (iii) holding an official athlete license issued by the Turkish Volleyball Federation. Participants were excluded if they had: (i) a history of knee joint injury; (ii) subjective knee pain with a numeric pain rating scale score greater than 3; (iii) any symptom or disorder of the lower extremity that could prevent jumping performance; (iv) marked static knee valgus; (v) a history of surgical intervention involving the knee joint; (vi) noticeable postural deformities in the foot; (vii) or if they were in the menstruation or follicular phases of their menstrual cycle (these participants were expected to be reassessed during the luteal phase) [20].

Based on the inclusion and exclusion criteria, the study included 18 athletes with DKV and 18 age-matched athletes without DKV.

### Outcome measures

#### Assessment of muscle biomechanical properties

The biomechanical properties of the muscles were evaluated using myotonometry, a non-invasive, hand-held device (MyotonPro^®^, Myoton AS, Tallinn, Estonia). This device provides objective measures of muscle tone, stiffness, and elasticity. Tone reflects the intrinsic tension of soft tissues at rest, stiffness represents resistance to deformation or elongation, and elasticity describes the ability of tissues to return to their original state after deformation [21]. The myotonometry method has shown good-to-excellent reliability, with intraclass correlation coefficients ranging from 0.69 to 0.98 [22].

The MyotonPro^®^ operates by delivering five brief (0.15 ms) mechanical impulses (0.56 N, 1 Hz) to the muscle surface and analyzing the resulting oscillation waves [23]. Device-specific algorithms calculate the following parameters:

- Tone (Hz): Oscillation frequency = (f_max_)
- Elasticity (%): Logarithmic decrement = ln (a_1_/a_3_)
- Stiffness (N/m): Dynamic stiffness = (a_max_ × m_probe_) / Δl

where *f*_*max*_ is the natural oscillation frequency, *ln* is the natural logarithm, *a*_*1*_ and *a*_*3*_ are the amplitudes of the first and third oscillation waves, *a*_*max*_ is the peak acceleration, *m*_*probe*_ is the probe mass, and *Δl* is the tissue deformation. Higher values indicate greater muscle tone and stiffness, and reduced elasticity [21].

**Fig. 2:**
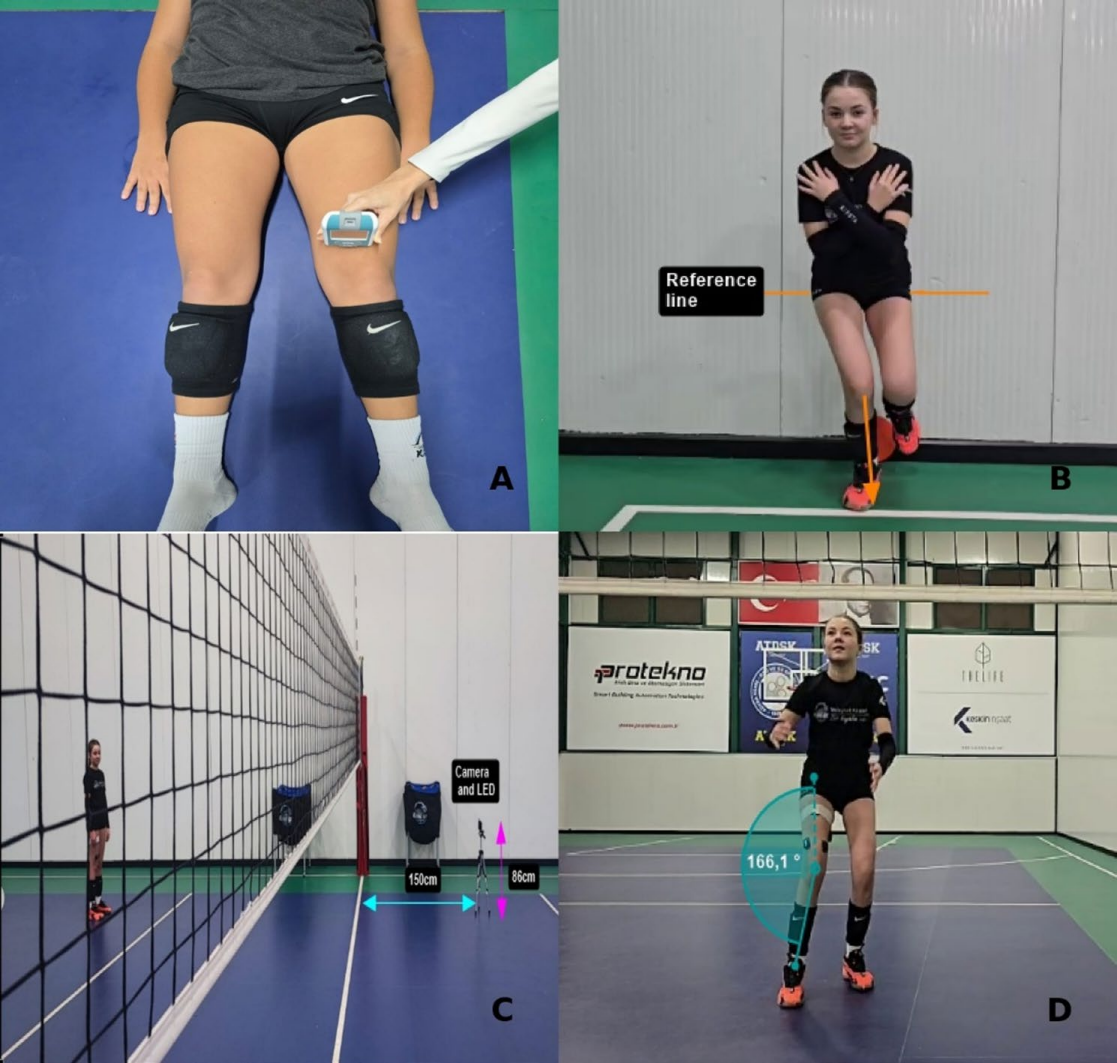
Outcome measures of the study. **A**: Assessment of thigh muscle biomechanical properties using myotonometry; **B**: Single leg squat test; **C**: Experimental setup showing camera positioning; **D**: Frontal-plane knee valgus angle measurement during the landing phase of the spike

Participants were instructed to avoid moderate-to-vigorous activity before testing. Measurements were performed on the gluteus medius (GM), adductor magnus (AM), vastus medialis (VM), vastus lateralis (VL), semimembranosus (HM), and biceps femoris (HL) of the dominant leg. The GM, AM, VM, and VL were assessed in a relaxed supine position, and the HM and HL were assessed in a relaxed prone position, all with the legs extended. The probe was positioned perpendicular to the muscle fibers at the thickest point of the muscles (Fig. 2A). For each muscle, three measurements were taken and averaged for analysis [18, 23].

#### Single leg squat test

The single-leg squat test (SLS) was adapted from the methods recommended by the National Academy of Sports Medicine [24]. The visual assessment of the SLS demonstrates moderate-to-good inter-rater reliability (pooled results of ICC/kappa: 0.58 (95% CI 0.50 to 0.65) and good-to-excellent intrarater reliability (pooled results of ICC/kappa: 0.68 (95% CI 0.60 to 0.74) [25]. Participants stood on the dominant leg with toes facing forward, while the non–weight-bearing leg was flexed. Hands were placed on the chest, and the head and eyes remained facing forward. Participants performed five consecutive SLS trials to a depth of 60° of knee flexion on the dominant leg. Before testing, 60° of knee flexion was measured for each participant using a goniometer. The corresponding height of the inferior gluteal contour at 60° was then marked on the wall adjacent to the participant. During the SLS trials, participants were instructed to descend until the inferior gluteal contour aligned with this reference mark. Each trial was visually observed by the researcher (V.D.). Participants were instructed to complete each trial in around 4 s, with 2 s allocated for the descent and 2 s for the ascent. To ensure temporal consistency, all participants were familiarized with the timing requirements prior to testing by practicing the procedure with a stopwatch (Fig. 2B).

Group assignment was based on real-time visual observation of knee kinematics. Participants were classified into the DKV (+) group if, in at least three of five trials, the midpoint of the patella moved medially past the great toe. Conversely, participants were placed in the DKV (-) group if, in at least three of five trials, the knee remained aligned with the hip and ankle throughout the movement [4].

#### 2D temporal and kinematic analysis

During the standardized volleyball spike (comprising a two-step approach, maximal vertical jump, and landing on the dominant leg - leg dominance was defined as the athlete’s preferred landing leg - ), the knee valgus angle in frontal plane and temporal intervals were assessed using two-dimensional (2D) video analysis. The 2D video analysis method has been shown to demonstrate high validity for knee valgus angle measurements (intraclass correlation coefficient: 0.72–0.91) [26].

Passive markers (0.5 cm in diameter) were bilaterally affixed with adhesive tape to the anterior superior iliac spine (ASIS), the midpoint of the patella, and the midpoint of the ankle joint. Each participant performed three trials, all of which were recorded for subsequent analysis. Video recordings of the spike were obtained with a high-resolution camera (GoPro Hero 11, California, USA) positioned 1.5 m in front of the volleyball net on a tripod at a height of approximately 86 cm (Fig. 2C). The camera was set at 240 fps and electronically synchronized with the EMG system via LED signaling.

Video recordings were analyzed using Kinovea software (version 0.8.25, Open Source Project) to determine the time intervals of the spike phases and to identify the maximal frontal-plane knee valgus angle occurring during landing. The spike movement was divided into four phases as follows: the approach phase, defined as the initial two-step running phase preceding the jump; the take-off phase, defined as the phase from the final foot contact after the approach to toe-off; the flight phase, defined as the airborne period between toe-off and initial ground contact; and the landing phase, defined as the interval from initial toe contact with the ground to the instant of maximum frontal-plane knee valgus angle. The time interval between initial toe contact during landing and the instant of maximal frontal-plane knee valgus angle was recorded, and only the EMG data obtained during this landing phase were analyzed. The knee valgus angle in frontal plane was calculated as the acute angle formed between the line ASIS and the midpoint of the patella, and the line connecting the patellar midpoint to the center of the ankle joint [4] (Fig. 2D). In addition, jump height, defined as the maximal vertical distance between the toe and the ground during flight, was also evaluated. Kinovea was calibrated for distance measurements according to each athlete’s leg length, which had been previously measured by the investigators.

#### Muscle activity

Muscle activity of the participants was measured during the execution of a standardized volleyball spike. EMG measurements were obtained from the dominant leg, which was the leg that made first contact during the landing. A wireless surface EMG system (TRIGNO, Delsys Inc., USA; input impedance < 10 Ω, baseline noise < 750 nV root mean square (RMS), effective EMG signal gain 909 V/V ± 5%) was used to record raw EMG activity from the GM, AM, VM, VL, HM, and HL muscles of the dominant leg. EMG signals were sampled at 2000 Hz with a bandwidth of 20–450 Hz, full-wave rectified, and subsequently smoothed using a second-order Butterworth low-pass filter with a 6 Hz cut-off frequency. The interelectrode distance was maintained at 1 cm. Skin preparation and electrode placement followed the recommendations of the Surface Electromyography for the Non-Invasive Assessment of Muscles (SENIAM) guidelines [27].

The mean RMS values obtained from each muscle during the landing phase (from initial toe contact to the instant of maximum frontal-plane knee valgus) were normalized to the peak EMG activation recorded during the landing phase of the spike (%maximum activation = muscle activity (RMS) / maximum activation of the respective muscle × 100). To minimize the risk of normalized EMG values exceeding 100%, a highly reliable normalization approach was employed (intraclass correlation coefficient > 0.80) [28, 29].

During the landing phase of the volleyball spike, the onset and offset times of muscle activation were determined. A muscle was classified as being “onset” when its EMG amplitude exceeded 3 standard deviations above the baseline signal within a 25-ms window, and as “offset” when the amplitude fell below this threshold [30]. EMG onset was defined as the first time point at which the signal exceeded the baseline threshold following the flight phase. Thus, onset timing reflected the re-initiation of muscle activation associated with landing rather than the initial activation of the muscle. For temporal reference, t = 0 ms was defined as the instant of initial foot contact during landing. The time difference between the onset of agonist and antagonist muscles associated with knee valgus control was subsequently calculated.

During the acceleration phase of the spike—spanning from the onset of the approach phase to the end of the take-off phase—thigh rotational velocity (deg/s) and acceleration (g) were measured using an inertial measurement unit (IMU) sensor (Avanti; Delsys Inc., Natick, MA, USA), which was temporally synchronized with the EMG system by the manufacturer and subjected to identical filtering procedures.The IMU measured triaxial acceleration (± 16 g range) and angular velocity (± 2000 deg/s range) at a sampling frequency of 2000 Hz. To minimize soft tissue oscillations, the sensor was firmly attached to the thigh with an adhesive band applied circumferentially around the limb [17] (Fig. 2D). Peak sagittal-plane rotational velocity and acceleration values were extracted for analysis [17, 18].

#### Data analysis

Considering the direction of tensile forces generated by the muscles and their anatomical functions, the AM, VM, and HM were defined as agonists, whereas the GM, VL, and HL were defined as antagonists for the medial knee displacement angle in the frontal plane. Ratios of muscle activity, tone, elasticity, and stiffness were subsequently calculated for AM/GM, VM/VL, and HM/HL [11, 18].

All statistical analyses were performed using the Statistical Package for the Social Sciences (SPSS, version 27.0; IBM Corp., Armonk, NY, USA). Graphs were generated with GraphPad Prism version 10.4.1 (GraphPad Software, San Diego, CA, USA). The distribution of the data was examined both visually (histograms and probability plots) and analytically (Shapiro–Wilk test). Normally distributed data were expressed as mean ± standard deviation (SD), non-normally distributed data as median [first quartile (Q1) – third quartile (Q3)], and categorical data as percentages (n, %). Between-group comparisons of sociodemographic, clinical, and outcome variables were performed using Pearson’s chi-square test, independent-samples t-test, or Mann–Whitney U test, as appropriate. In addition, effect sizes were calculated for continuous variables using Cohen’s d, with values of approximately 0.20, 0.50, and 0.80 interpreted as small, medium, and large effects, respectively.

Parameters showing a significant correlation at *p* < 0.15 with knee valgus angle were subsequently included in a two-model multiple linear regression analysis to identify determinants of knee valgus angle. Prior to model fitting, assumptions of multiple regression were verified, including normal distribution of residuals, homoscedasticity, absence of multicollinearity (variance inflation factor, VIF < 10), independence of errors (Durbin–Watson statistic), and linearity between predictors and the outcome variable. Participants’ demographic and sport-specific characteristics, jump height and thigh rotational acceleration and velocity during the approach to take-off interval were considered as potential confounders. A p value < 0.05 was considered statistically significant.

## Results

**Fig. 3:**
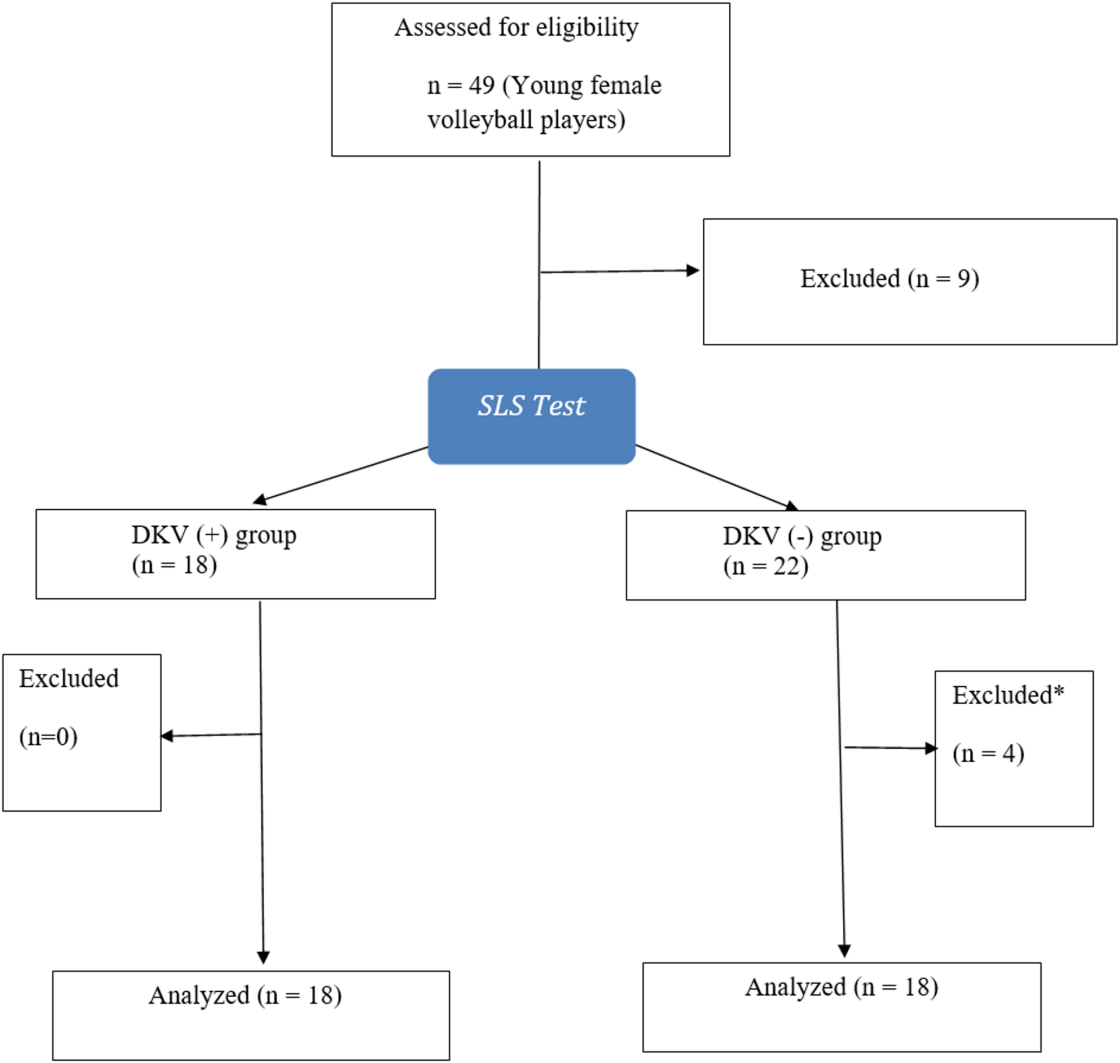
Flow chart of the participants. (*): Not matched with any participant in the DKV (+) group in terms of age. Abbreviations: DKV, Dynamic knee valgus; SLS: Single-leg squat test

Of the 49 athletes initially screened for eligibility, 18 who met the inclusion criteria and demonstrated DKV (+) on the SLS test were assigned to the DKV (+) group. Nine athletes were excluded for various reasons: history of knee surgery (*n* = 1), previous knee injury (*n* = 3), static knee valgus > 15° (*n* = 1), and declined to participate (*n* = 4). From the 22 athletes without DKV, 18 were selected to form the DKV (–) group based on age-matching with the DKV (+) athletes, and their data were included in the analysis (Fig. 3).

No significant differences were observed between the DKV (+) group and the DKV (-) in terms of age, height, weight, body mass index, volleyball experience, or playing positions (*p* > 0.05). Kinematic analysis of the spike revealed that rotational velocity and accelaration of the approach phase and jump height did not differ significantly between the groups (*p* > 0.05). Accordingly, these findings indicate that potential confounders were comparable between the two groups. However, the DKV (+) group exhibited significantly greater knee valgus angles in frontal plane during landing phase compared to the control group (*p* = 0.045, Cohen’s d = 0.72 – medium effect size –) (Table 1).

**Table 1:** Demographic, sport-specific, and spike-related kinematic characteristics of the DKV(+) and DKV(–) groups

Variable	DKV (+) group (*n* = 18)	DKV (-) group (*n* = 18)	Mean difference (95% CI)	Effect size (Cohen’d or Cramer’s V)	*p*-value
Age (years)^b^	13.0 (12.0–17.0)	13.0 (12.0–16.0)	0.5 (-0.5 to 1.5)	0.35	0.293^Ω^
Height (cm)^a^	160.5 ± 7.9	160.9 ± 7.7	-0.5 (-5.8 to 4.8)	0.06	0.855^Ϯ^
Weight (kg)^b^	50.8 (37.8–85.4)	49.6 (39.0–75.0)	-0.7 (-8.9 to 7.4)	0.06	0.782 ^Ω^
BMI (kg/m²)^b^	19.6 (15.4–26.7)	19.0 (16.1–24.4)	-0.3 (-2.4 to 1.8)	0.09	0.561 ^Ω^
Sports experience (years)^b^	3.0 (2.0–7.0)	3.0 (2.0–8.0)	-0.1 (-0.6 to 0.5)	0.07	0.846 ^Ω^
Playing position, n (%)			NA	0.43	0.153^Ψ^
Outside hitter	9 (50)	5 (27.8)			
Middle blocker	4 (22.2)	6 (33.3)			
Setter	3 (16.7)	5( 27.8)			
Libero	2 (11.1)	2 (11.1)			
Maximum velocity of the approach-take-off phase (deg/s)^b^	574.0 (426.3 - 805.78)	516.5 (443.3–690.5)	71.4 (-108.8 to 251.7)	0.23	0.506 ^Ω^
Maximum accelaration of the approach-take-off phase (g)^a^	8.93 ± 3.72	8.08 ± 3,88	0.9 (-1.7 to 3.4)	0.27	0.502^Ϯ^
Jump height (cm)^a^	32.9 ± 6.3	32.88 ± 8.48	0.1 (-5.0 to 5.1)	0.01	0.983 ^Ϯ^
Knee valgus angle during spike, (deg)^b^	13.8 (9.7–19.0)	9.9 (5.9–14.7)	4.1 (0.2 to 8.3)	0.72	0.045 ^Ω^

a: mean ± standard deviation, b: median (25% – 75%), (Ϯ): Unpaired t test, (Ω): Mann – Whitney U test, (Ψ): Pearson’s chi square test

*Abbreviations*: *DKV* Dynamic knee valgus, *BMI* Body mass index

**Fig. 4:**
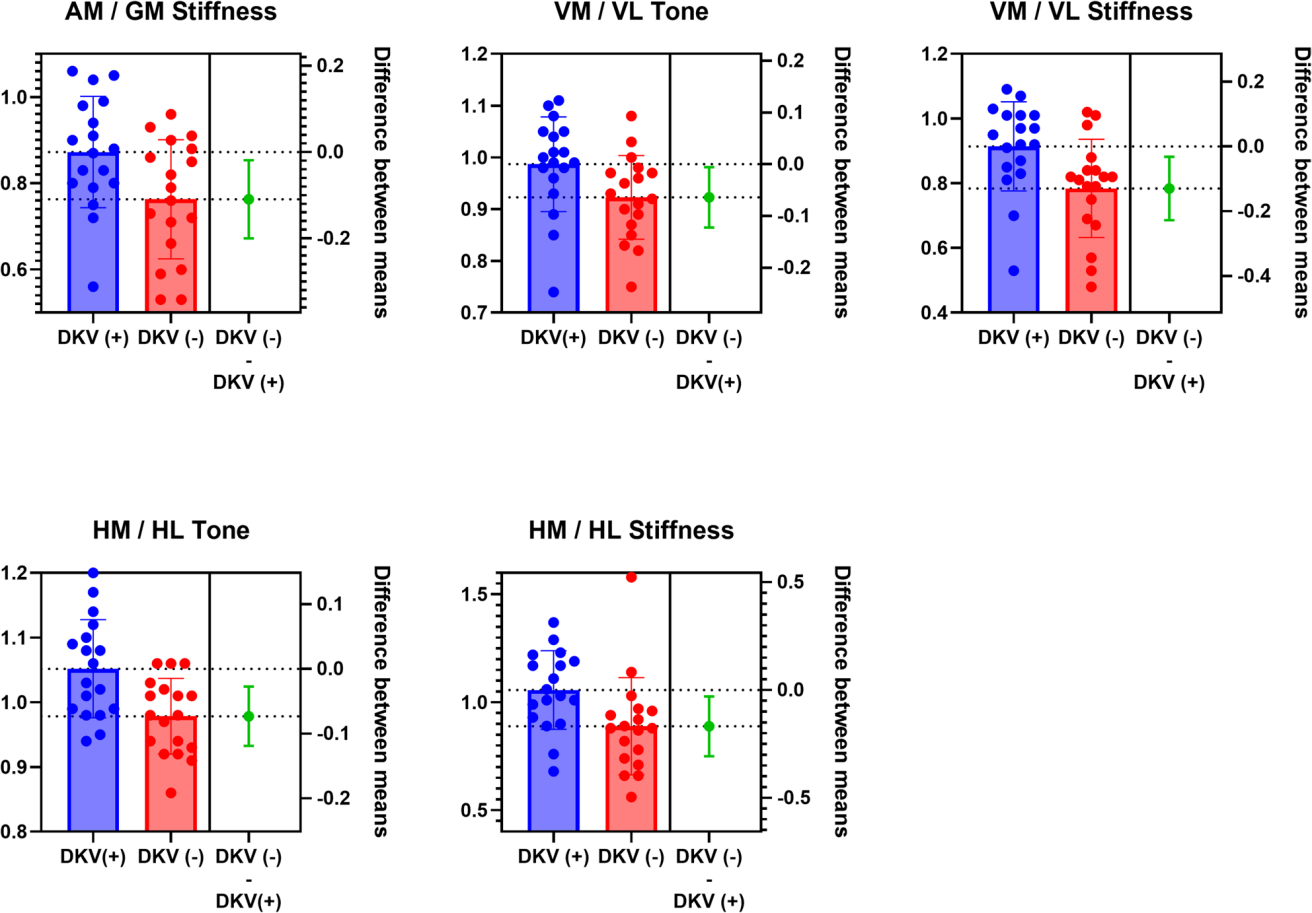
Muscle tone and stiffness ratios showing significant differences between the DKV(+) and DKV(–) groups. Abbreviations: GM, Gluteus medius; AM, Adductor magnus; VM, Vastus medialis; VL, Vastus lateralis; HM, semimembranosus; HL, biceps femoris; DKV, Dynamic knee valgus

Myotonometric assessments showed that VM/VL and HM/HL tone ratios, as well as AM/GM, VM/VL, and HM/HL stiffness ratios, were significantly elevated in the DKV (+) group (*p* < 0.05), while no group differences were observed in muscle elasticity (*p* > 0.05). The between-group differences demonstrated medium-to-large effect sizes (Cohen’s d = 0.71–1.07 – medium-to-large effect sizes –). (Table 2; Fig. 4).

**Table 2:** Comparison of muscle tone, elasticity, and stiffness of lower limb muscles between the DKV(+) and DKV(–) groups

Variable	TONE (Natural Oscillation Frequency [Hz])			ELASTICITY (Logarithmic Decrement [relative unit])			STIFFNESS (Dynamic Stiffness [*N*/m])		
	DKV (+) group (*n* = 18)	DKV (-) group (*n* = 18)	*p*-value/ Cohen’s d	DKV (+) group (*n* = 18)	DKV (-) group (*n* = 18)	*p*-value/ Cohen’s d	DKV (+) group (*n* = 18)	DKV (-) group (*n* = 18)	*p*-value/ Cohen’s d
GM	14.3 ± 1.2	14.5 ± 1.5	0.712 / 0.13	0.95 ± 0.22	0.88 ± 0.23	0.352 / 0.32	184.6 ± 33.1	197.1 ± 47.4	0.372 / 0.31
AM	11.9 ± 0.9	11.4 ± 0.8	0.110 / 0.55	0.96 ± 0.30	0.88 ± 0.18	0.330 / 0.33	164.3 ± 26.7	148.3 ± 22.3	0.059 / 0.65
VM	13.7 ± 1.4	13.3 ± 1.1	0.372 / 0.30	1.03 ± 0.12	0.94 ± 0.15	0.064 / 0.64	210.6 ± 39.5	192.5 ± 34.2	0.151 / 0.49
VL	13.9 ± 1.1	14.5 ± 1.4	0.187 / 0.45	1.07 ± 0.14	0.96 ± 0.25	0.138 / 0.51	231.9 ± 35.7	252.9 ± 57.7	0.198 / 0.44
HM	14.5 ± 1.4	14.1 ± 1.2	0.318 / 0.34	0.90 ± 0.11	0.88 ± 0.14	0.482 / 0.24	222.8 ± 55.1	199.6 ± 36.3	0.143 / 0.50
HL	13.9 ± 1.5	14.5 ± 1.6	0.273 / 0.37	0.96 ± 0.14	0.88 ± 0.14	0.124 / 0.53	216.1 ± 57.2	233.6 ± 58.2	0.369 / 0.36
AM/GM	0.84 ± 0.07	0.80 ± 0.08	0.130 / 0.53	1.01 ± 0.39	1.04 ± 0.39	0.895 / 0.06	0.87 ± 0.18	0.76 ± 0.14	0.040 / 0.71
VM/VL	0.99 ± 0.09	0.92 ± 0.08	0.036 / 0.73	0.98 ± 0.14	0.96 ± 0.15	0.686 / 0.14	0.91 ± 0.14	0.78 ± 0.15	0.011 / 0.90
HM/HL	1.05 ± 0.07	0.98 ± 0.06	0.003 / 1.07	0.97 ± 0.18	1.00 ± 0.15	0.580 / 0.19	1.06 ± 0.18	0.89 ± 0.23	0.019 / 0.82

*Abbreviations*: *GM* Gluteus medius, *AM* Adductor magnus; *VM* Vastus medialis, *VL* Vastus lateralis, *HM* Semimembranosus, *HL* Biceps femoris, *DKV* Dynamic knee valgus

**Fig. 5:**
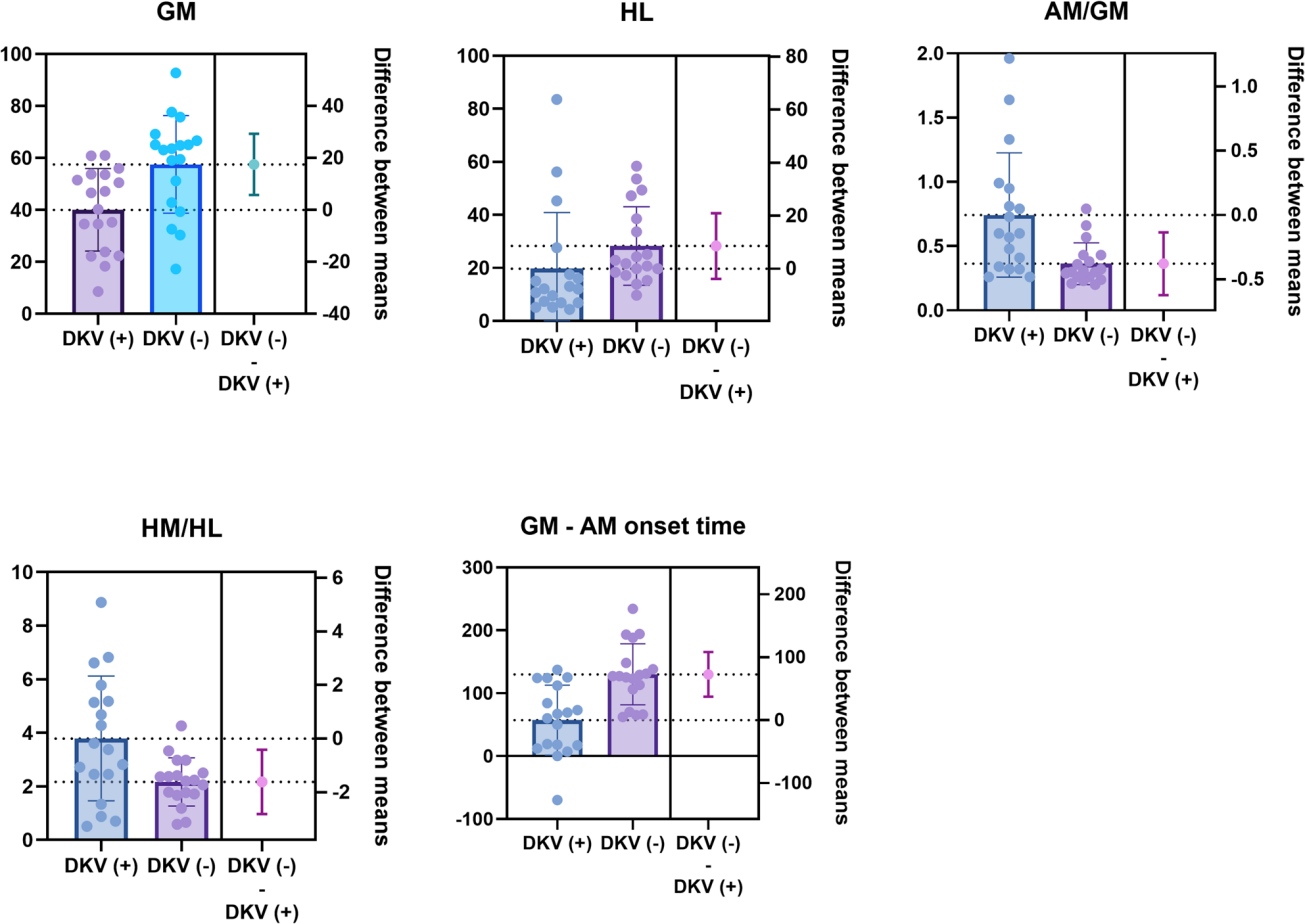
Significant between-group differences in normalized EMG (% of maximum activation) and onset time. Abbreviations: GM, Gluteus medius; AM, Adductor magnus; HM, Semimembranosus; HL, Biceps femoris; DKV, Dynamic knee valgus

According to EMG analysis, the DKV (+) group demonstrated significantly lower activation in the GM and HL muscles, along with higher AM/GM and HM/HL ratios. Additionally, the GM-AM onset time difference was significantly shorter in the DKV (+) group (p < 0.05 for all and with high effect sizes – 1.00 to 1.39 – ) (Table 3; Fig. 5).

**Table 3:** Comparison of normalized EMG activation levels and onset times during landing between the DKV(+) and DKV(–) groups

% (Maximum activation)	DKV (+) group (*n* = 18)	DKV (-) group (*n* = 18)	Mean difference (95% CI)	Effect size (Cohen’s d)	*p*-value
GM^a^	40.1 ± 15.8	57.5 ± 18.8	-17.5 (-29.3 to -5.7)	1.00	0.005
AM^b^	19.4 (14.9–40.5)	16.7 (13.6–26.3)	9.4 (-2.3 to 21.1)	0.34	0.311
VM^a^	50.9 ± 24.0	56.6 ± 17.5	-5.7 (-19.9 to 8.6)	0.27	0.426
VL^b^	14.6 (9.3–37.2)	28.6 (18.5–37.8)	-8.8 (-19.8 to 2.3)	0.62	0.076
HM^a^	45.8 ± 16.0	54.8 ± 23.6	-9.0 (-22.8 to 4.7)	0.45	0.189
HL^b^	12.0 (6.9–20.0)	22.2 (18.1–40.6)	-8.6 (-21.0 to 3.8)	1.04	0.005
AM/GM^b^	0.6 (0.3–0.9)	0.3 (0.2–0.4)	0.4 (0.1 to 0.6)	1.05	0.001
VM/VL^b^	2.3 (1.1–4.7)	2.1 (1.6–2.7)	0.6 (-0.1 to 2.4)	0.19	0.569
HM/HL^b^	3.5 (2.1–5.9)	2.2 (1.7–2.6)	2.2 (0.4 to 3.9)	0.89	0.015
Difference between onset time					
GM - AM^a^	57.1 ± 55.7	129.9 ± 48.6	-72.8 (-108.3 to -37.4)	1.39	< 0.001
VL - VM^b^	118.0 (86.8–25.2)	119.0 (52.5–123.0)	-20.2 (-46.7 to 6.2)	0.34	0.311
HL - HM^b^	113.0 (9.2–127.7)	111.5 (60.5–121.5)	2.3 (-35.5 to 40.2)	0.01	0.975

a: mean ± standard deviation, b: median (25% – 75%)

*Abbreviations:* *GM* Gluteus medius, *AM* Adductor magnus, *VM* Vastus medialis, *VL* Vastus lateralis, *HM* Semimembranosus, *HL* Biceps femoris

Correlation analysis showed no significant associations between muscle activity, the onset time differences of agonist–antagonist muscles or muscle biomechanical properties and knee valgus angle. However, GM–AM onset time, HM elasticity, and HM/HL tone demonstrated correlations with knee valgus angle at the p < 0.150 level and were therefore included in the multiple regression analysis (Table 4).

**Table 4:** Correlations between knee valgus angle during landing with EMG, onset time, and muscle biomechanical properties

	Correlation for EMG and KVA		Correlation for onset time and KVA		Correlation for tone and KVA		Correlation for elasticity and KVA		Correlation for stiffness and KVA	
	*r*	*p*-value	*r*	*p*-value	*r*	*p*-value	*r*	*p*-value	*r*	*p*-value
GM	-0.193	0.260	NA	NA	-0.017	0.922	0.095	0.587	-0.136	0.435
AM	0.009	0.960	NA	NA	0.102	0.555	-0.051	0.768	-0.078	0.651
VM	0.159	0.356	NA	NA	0.231	0.176	0.069	0.691	0.115	0.504
VL	-0.056	0.748	NA	NA	-0.192	0.261	-0.160	0.353	-0.022	0.897
HM	0.154	0.356	NA	NA	-0.021	0.904	0.268	0.114	0.133	0.439
HL	-0.194	0.256	NA	NA	-0.174	0.310	-0.217	0.204	-0.042	0.806
AM/GM	0.191	0.266	0.279	0.099	0.104	0.551	0.073	0.677	0.146	0.403
VM/VL	0.096	0.579	0.015	0.930	0.002	0.992	0.071	0.680	-0.022	0.899
HM/HL	0.071	0.681	0.017	0.920	-0.258	0.142	-0.053	0.761	-0.104	0.547

*Abbreviations*: *GM* Gluteus medius, *AM* Adductor magnus, *VM* Vastus medialis, *VL* Vastus lateralis, *HM* Semimembranosus, *HL* Biceps femoris, *KVA* Knee valgus angle

The multiple linear regression analysis did not identify any statistically significant predictors of knee valgus angle during the landing phase of the spike. In Model 1, the GM–AM onset time showed a negative but non-significant association with medial knee displacement angle (β = − 0.28, p = 0.099). In Model 2, after additionally including HM elasticity and HM/HL tone, GM–AM onset time (β = − 0.33, p = 0.092) and HM elasticity (β = 0.32, p = 0.095) demonstrated trends toward significance, but did not reach the p < 0.05 threshold. HM/HL tone was not associated with knee valgus angle (p = 0.882). The overall explanatory power of the models was low (Adjusted R² = 0.08 for Model 1; ΔR² = 0.09 for Model 2) (Table 5).

**Table 5:** Determinants of knee valgus angle during the landing phase of the spike

	B (SE)	95% CI for B	Beta	*p*-value
Model 1				
AM – GM onset time	0.03 (0.02)	-0.01 to 0.07	0.28	0.099
(Constant (SE) = 15.80 (2.10), Adjusted R^2^ = 0.08)				
Model 2				
AM – GM onset time	0.04 (0.02)	-0.01 to 0.08	0.33	0.092
HM elasticity	17.77 (10.31)	-3.28 to 38.85	0.32	0.095
HM / HL tone	2.86 (1.88)	-41.88 to 36.16	0.03	0.882
(Constant (SE) = 3.39 (1.82), Delta R^2^ = 0.09)				

*Abbreviations*: *GM* Gluteus medius, *AM* Adductor magnus, *HM* Semimembranosus, *HL* Biceps femoris *B *unstandardized coefficients, *SE* Standard error β standardized coefficients

The stepwise multiple linear regression analysis demonstrated that potential confounders had no significant effect on the knee valgus angle. Therefore, no adjustments were made for any of the potential confounders.

## Discussion

This study aimed to compare the biomechanical properties of muscles in young female volleyball players with and without DKV. The results indicated that the VM/VL and HM/HL ratios for both muscle tone and stiffness were higher in the DKV (+) group compared to the DKV (−) group. Similarly, the AM/GM ratio for muscle stiffness was also elevated in athletes with DKV (+). However, neither the biomechanical properties nor the activation levels of the assessed muscles demonstrated a significant association with the frontal plane valgus angle observed during the landing phase of the spike. To the best of our knowledge, this is the first study to examine the relationship between DKV and the biomechanical properties of muscles specifically in volleyball athletes, thereby offering a novel contribution to the existing body of literature.

The mechanical properties of muscles are fundamental determinants of body segment alignment and movement characteristics in both static and dynamic conditions [17, 18, 23]. Deniz and Kılcı demonstrated that muscle elasticity is an important factor determining both ball and leg velocity during soccer kick, which is a complex dynamic activity [17]. In a recent study that is more relevant to the topic of the present research, it was reported that in athletes with scapular dyskinesis, not only alterations in muscle activation patterns but also changes in muscle biomechanical properties contribute to the disruption of scapular movement patterns [18]. Findings from previous studies indicate that muscle biomechanical properties may need to be considered—alongside muscle activation patterns—when discussing potential contributors to DKV. In contrast to these, a previous study investigating the relationship between DKV and the biomechanical properties of muscles in healthy adults reported no significant association between these two parameters [19]. Our study demonstrated results similar to those of Llurda-Almuzara et al. (2021) when the interactions between muscles and their antagonists were not taken into account. However, when agonist/antagonist muscle ratios were taken into account, it was observed that the tone and stiffness of muscles known to contribute to DKV during high contraction patterns were elevated in athletes with DKV (+). Given that DKV arises not from the isolated biomechanical properties of individual muscles but from their functional interactions with antagonistic muscle groups [4], these findings align with the broader literature established by previous EMG studies [11, 31].

In our study, increased stiffness and tone observed in the medial knee and hip muscles of athletes with DKV may be attributed to greater neuromuscular activation of these muscles during sport-specific movements [32, 33]. Volleyball-specific movements such as jumping, landing, and receiving the ball by squating may involve greater eccentric and concentric activation of the medial knee and hip muscles compared with their antagonists in athletes exhibiting DKV. Chronic exposure to high-load, repetitive activation patterns is likely to lead to adaptive increases in passive muscle stiffness and resting tone, indicating a long-term neuromechanical adaptation rather than a pathological dysfunction [34, 35]. The lack of a significant relationship or effect between the frontal plane valgus angle observed during the landing phase of the spike and the biomechanical properties of the muscles further supports this interpretation.

In the current study, although tone and stiffness differed between groups, elasticity did not. This may be explained by the fact that tone and stiffness are more sensitive to neuromuscular loading patterns and chronic activation differences, whereas elasticity reflects deeper viscoelastic tissue characteristics that change more slowly and are less affected by sport-specific asymmetrical demands [36]. Furthermore, elasticity measurements generally show greater variability and lower discriminatory sensitivity compared with stiffness [36], which may also contribute to the absence of significant between-group differences.

In the present study, IMU-derived rotational velocity and acceleration were analyzed exclusively during the approach-to–take-off interval and treated as potential confounding factors. Unlike previous studies that assessed landing mechanics from a static starting position or standardized drop heights [7–10], our protocol involved a sport-specific spike movement in which approach-take off speed and jump height could vary between athletes. Since these factors may influence neuromuscular activation and landing mechanics, they were recorded and compared between groups. This allowed us to interpret the observed EMG values during landing independently of approach-take off speed and jump height.

One of the noteworthy findings of our study is the absence of a significant relationship between muscle activation and the valgus angle observed during the landing phase of the spike in athletes with DKV (+). Previous research has suggested that DKV is associated with reduced activation or delayed recruitment of the gluteal muscles [31, 37]. A possible explanation for this discrepancy is that DKV was identified using a single-leg squat task, while EMG measurements were obtained during the spike movement. The kinematic demands of these two tasks differ substantially, which may explain the lack of observed association [38]. Furthermore, testing was conducted under relatively controlled conditions, free from external perturbations and the situational demands commonly encountered during matches or high-intensity training. This controlled environment may have facilitated a more stable landing strategy, thereby reducing the expression of valgus alignment that is typically observed under real-world competitive conditions [39].

EMG onset should not be interpreted as the initial activation of the muscle during the spike, but rather as the re-initiation of muscle activity specifically associated with the landing preparation phase [40]. Given that spike execution involves continuous multi-joint activation throughout the approach and take-off phases, the detected onset reflects a transition in neuromuscular strategy. Landing represents a distinct neuromotor task within the spike sequence, requiring rapid reorganization of motor output to ensure joint stabilization and impact attenuation. Accordingly, the onset identified approximately 50–150 ms prior to initial foot contact is best interpreted as a feedforward anticipatory activation aimed at increasing joint stabilization and optimizing frontal-plane control before ground impact [41]. Nevertheless, the regression analysis demonstrated that muscle onset timing was not significantly associated with DKV. This finding suggests that onset timing alone does not directly determine frontal-plane knee mechanics, and that the observed timing differences may instead reflect a task-specific neuromuscular adaptation within a complex, multi-muscle control strategy rather than a primary mechanical driver of DKV.

## Limitations

The present study has several limitations. Firstly, although the knee valgus angle was assessed using a validated 2D analysis method [26], this technique captures only frontal plane motion and does not account for transverse plane rotations, which would require 3D motion analysis [42]. Secondly, DKV was identified during the SLS, whereas EMG measurements were obtained during the spike movement, potentially introducing task-specific kinematic variability. Thirdly, menstrual cycle phase was determined using a calendar-based method. The expected hormonal status of the participants was estimated according to the day of their cycle rather than confirmed through biochemical testing. Therefore, the inability to objectively verify hormonal levels—and the potential influence of estrogen fluctuations on knee laxity—should be considered a limitation of the study. Fourthly, intra-session reliability metrics (ICC or CV%) for the SLS assessment, myotonometric measurements, and EMG amplitude were not calculated in this study. Fifthly, while the applied EMG normalization approach was selected for dynamic task specificity rather than to prevent values exceeding 100%, its validity for inter-muscle comparisons is limited and the findings should be interpreted cautiously. Finally, the relatively small sample size and the inclusion of only adolescent female volleyball players limit the generalizability of the findings to broader athletic populations

## Conclusion

In this study, athletes with DKV demonstrated higher medial-to-lateral muscle tone (VM/VL and HM/HL) and stiffness ratios (VM/VL, HM/HL, and AM/GM) compared with those without DKV. However, these biomechanical properties, along with muscle activation levels, did not significantly influence the frontal-plane knee valgus angle during the landing phase of the volleyball spike. Future research is warranted to determine whether these biomechanical alterations reflect a functional adaptation or constitute a potential risk factor for knee dysfunction. 

## Data Availability

The data that support the findings of this study are available from the corresponding author upon reasonable request.

## Abbreviations

DKV: Dynamic knee valgus
GM: Gluteus medius
AM: Adductor magnus
VM: Vastus medialis
VL: Vastus lateralis
HM: Semimembranosus
HL: Biceps femoris
SLS: Single-leg squat test
ASIS: Anterior superior iliac spine
RMS: Root mean square
IMU: Inertial measurement unit
EMG: Electromyography
